# Interfering with stem cell-specific gatekeeper functions controls tumour initiation and malignant progression of skin tumours

**DOI:** 10.1038/ncomms6874

**Published:** 2015-01-22

**Authors:** Monika Petersson, Karen Reuter, Heike Brylka, Andreas Kraus, Peter Schettina, Catherin Niemann

**Affiliations:** 1 Center for Molecular Medicine Cologne, University of Cologne, Robert-Koch-Strasse 21, 50931 Cologne, Germany; 2 Center for Biochemistry, Medical Faculty, University of Cologne, 50931 Cologne, Germany

## Abstract

Epithelial cancer constitutes a major clinical challenge and molecular mechanisms underlying the process of tumour initiation are not well understood. Here we demonstrate that hair follicle bulge stem cells (SCs) give rise to well-differentiated sebaceous tumours and show that SCs are not only crucial in tumour initiation, but are also involved in tumour plasticity and heterogeneity. Our findings reveal that SC-specific expression of mutant Lef1, which mimics mutations found in human sebaceous tumours, drives sebaceous tumour formation. Mechanistically, we demonstrate that mutant Lef1 abolishes p53 activity in SCs. Intriguingly, mutant Lef1 induces DNA damage and interferes with SC-specific gatekeeper functions normally protecting against accumulations of DNA lesions and cell loss. Thus, normal control of SC proliferation is disrupted by mutant Lef1, thereby allowing uncontrolled propagation of tumour-initiating SCs. Collectively, these findings identify underlying molecular and cellular mechanisms of tumour-initiating events in tissue SCs providing a potential target for future therapeutic strategies.

## Abstract (web summary)

The presence of multiple stem and progenitor cells in the skin has a major impact on the formation of different epidermal tumours. Here the authors identify bulge stem cells as the cells of origin of sebaceous tumours through genetic lineage tracing and molecular studies in a mouse model.

## Graphical abstract

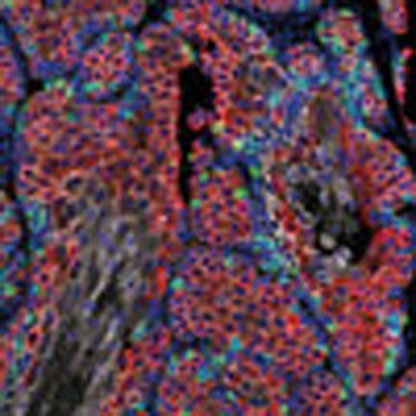

Epithelial skin tumours are generally heterogeneous cell populations embedded in a unique microenvironment^1^,^2^. Tumour growth is driven by tumour-initiating cells carrying mutations that favour cell expansion and escape normal growth control crucial for tissue homoeostasis^3^,^4^. The underlying molecular mechanisms allowing these cells to respond with tumour initiation are not well understood.

The skin epithelium generates various types of tumours. Multiple stem and progenitor cell populations ensure maintenance of the interfollicular epidermis (IFE) and its appendages, including hair follicles (HFs) and sebaceous glands (SGs) under homoeostatic conditions and therefore could contribute differently to this tumour heterogeneity^5^,^6^. Major pools of stem cells (SCs) localize to the bulge, a region in the lower permanent part of the HF, the isthmus region (IR) of the HF and the junctional zone (JZ) where the SG attaches to the HF (Fig. 1a). Multipotent SCs of the bulge are characterized by expression of distinct marker molecules, including Keratin 15 (K15), Sox9, NFATc1 and others and have been isolated on the basis of high expression of CD34 and integrin α6 (CD34^+^/Itga6^high^)^7^,^8^,^9^,^10^,^11^,^12^,^13^,^14^. In addition, the IR just above the HF bulge comprises stem and progenitor pools, which are positive for Lgr6 and the MTS24 antigen Plet1 (refs 15, 16, 17). Furthermore, lineage-restricted SCs residing in the JZ have been isolated based on the expression of Lrig1 and are involved in the regeneration of the IFE and the SG^18^,^19^. However, the specific role of these diverse SC compartments in the process of tumour formation and maintenance is not well understood.

Recently, multiple epidermal cell populations have been tested for their potential to initiate tumour growth in mouse models for different types of skin cancer including squamous cell carcinoma (SCC) and basal cell carcinoma (BCC). These studies have identified different HF progenitor pools as cellular source for SCC and potentially BCC formation^2^,^20^,^21^,^22^,^23^,^24^,^25^,^26^,^27^. However, a cell-of-origin for sebaceous types of skin cancer as well as mechanisms of initiation and progression of sebaceous tumours are not known. Sebaceous skin tumours comprise a range of epidermal lesions from sebaceous hyperplasia, benign adenoma and sebaceoma to malignant sebaceous carcinoma^28^. The underlying molecular mechanisms resulting in the generation of sebaceous lesions are not sufficiently understood. With this regard, mutations within the β-catenin-binding domain of the transcription factor Lef1, an important mediator of canonical Wnt signalling, have been identified in human sebaceous tumours^29^. It has been demonstrated that these mutations result in decreased canonical Wnt signalling activity and the repression of Wnt/β-catenin target gene expression^29^.

Previously, we have established a robust *in vivo* mouse model to induce well-differentiated sebaceous adenomas in a spatially and temporally controlled manner^30^. In this mouse model, N-terminally deleted Lef1 is expressed under the control of the keratin 14 promoter (K14ΔNLef1) thereby targeting the mutant transcription factor to the basal keratinocytes of the IFE, the HFs and SGs^31^. K14ΔNLef1 mice mimic the phenotype of human Lef1 mutations and develop spontaneous sebaceous adenomas^29^,^30^,^31^.

Here we use this inducible tumour model to explore whether multipotent bulge SCs are involved in tumour initiation and examine the potential role of lineage-restricted stem and progenitor cells in propagation and progression of epithelial tumours *in vivo*. One approach to address this issue of cell-of-origin is lineage tracing of individually labelled cells in the process of tumour formation^32^. This experimental strategy allows not only to investigate the competence of cells to initiate cancer as a consequence of accumulation of relevant mutations but importantly, to determine the contribution of a distinct cell type to early stages of tumour development under physiological conditions. Our experiments address the role of mutant Lef1 in tumour initiation specifically by SCs. This study led to the identification of the underlying molecular and cellular mechanisms of SC-driven skin tumour initiation, thereby providing a potential strategy to test for novel and effective therapies to treat epidermal tumours.

## Results

### Bulge SCs give rise to differentiated sebaceous skin tumours

**Figure 1:**
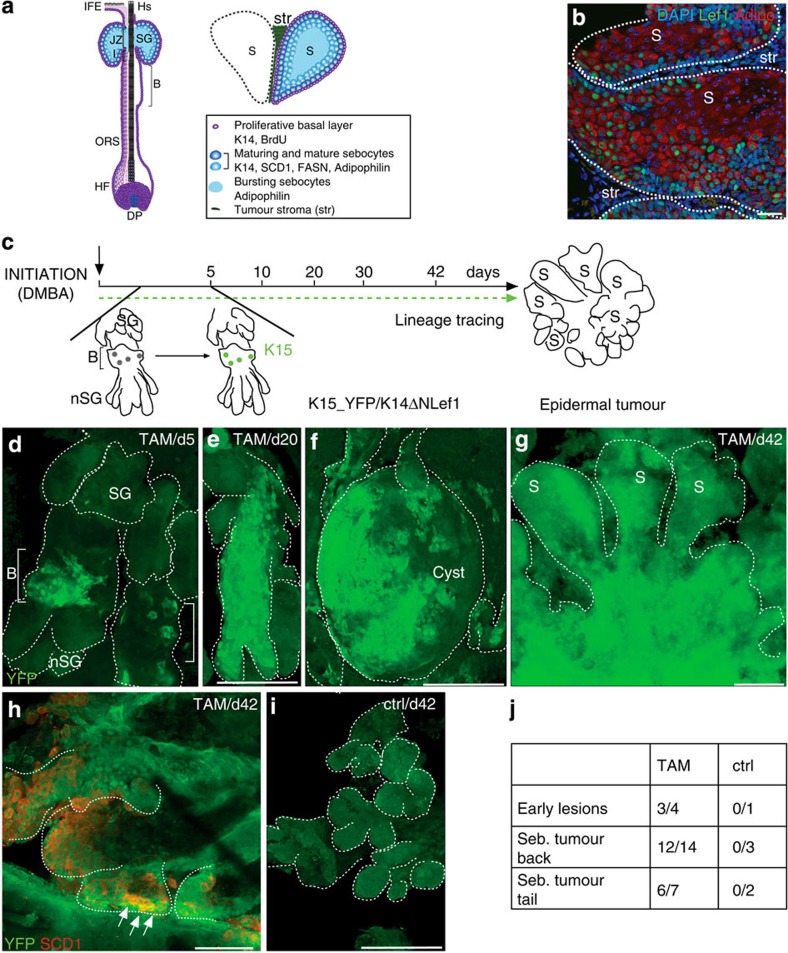
Bulge SCs constitute a cell-of-origin for differentiated sebaceous skin tumours. (**a**) Architecture and marker expression within the SG of the pilosebaceous unit and lobules of sebaceous adenoma. B, bulge; BrdU, 5-bromo-2′-deoxyuridine; FASN, fatty acid synthetase; HF, hair follicle; HS, hair shaft; I, isthmus; IFE, interfollicular epidermis; JZ, junctional zone; K14, keratin 14; ORS, outer root sheath; S, sebaceous tumour lobule; SCD1, stearoyl coenzyme A desaturase; SG, sebaceous gland; str, tumour stroma. (**b**) Immunofluorescence staining of sebaceous tumour lobules of K14ΔNLef1 transgenic mice for expression of mutant Lef1 (green) and differentiation marker Adipophilin (red). (**c**) Experimental strategy to trace progeny of K15-derived bulge stem cells (BSCs) during epidermal tumorigenesis. (**d**) Detection of YFP^+^ SCs within the bulge of deformed HF 5 days following TAM application. nSG, *de novo* sebaceous gland. (**e**–**g**) Clonal expansion of YFP^+^ SC progeny in epidermal whole mounts of early lesions (cysts) and within the tumour lobules 42 days following DMBA treatment. (**h**) Co-localization of YFP- and SCD1 (red)-expressing cells in tumours (arrows). (**i**) Control tissue remains negative for YFP. ctrl, oil treatment. (**j**) Quantification of YFP^+^ tumours at different stages of development and from different body sites. Scale bars, 50 μm.

In a first set of experiments, we performed genetic lineage tracing experiments to investigate whether HF SCs from the bulge region are capable of generating well-differentiated tumours. To trace the fate of bulge SC clones at distinct steps of tumour formation, we employed an inducible and robust murine tumour model for sebaceous adenomas^29^,^30^,^31^. Particularly, treatment of mice expressing mutant Lef1 in the basal compartment of the epidermis (K14^+^) (K14ΔNLef1 mice) with a single sub-threshold dose of the carcinogen DMBA (150 nM, 7,12-dimethylbenz(a)anthracene) induced the formation of sebaceous tumours in high frequency in a spatially and temporally controlled manner (Fig. 1a–c and Supplementary Fig. 1a–c). Sebaceous adenomas exhibit a distinct lobular architecture and are populated by highly differentiated and mature sebocytes. Proliferation of tumour cells takes place in the outer basal layer at the periphery of tumour lobules strongly resembling the natural regeneration process taking place during SG homeostasis (Fig. 1a–c and Supplementary Fig. 1a). Cells leaving the basal compartment undergo a defined programme of differentiation and express marker molecules including stearoyl coenzyme A desaturase1 (SCD1), adipophilin and fatty acid synthetase (FASN), which are also expressed by sebocytes of normal SGs^33^,^34^. Thus, the typical SG architecture and hierarchy of undifferentiated and mature sebocytes are maintained in well-differentiated sebaceous adenoma (Fig. 1a,b).

To test if HF SCs give rise to sebaceous tumours in their natural environment, K14ΔNLef1 transgenics were crossed to K15CreER(G)^T2^ and Rosa26REYFP (R26YFP) mice (K15_YFP/K14ΔNLef1), which allow fate mapping of individual bulge SC descendants^35^,^36^. Progeny of YFP-labelled bulge cells was traced over time by analysing epidermal whole mounts harvested from K15_YFP/K14ΔNLef1 mice at different time points following DMBA (150 nM) and tamoxifen (TAM) treatment (Fig. 1c–h). As shown previously, TAM treatment of K15CreER(G)^T2^ mice results in Cre activation specifically in SCs of the HF bulge region and bulge SCs are also marked in deformed HF of K14ΔNLef1 mice but not in control (oil-treated) mice of the same genotype (Fig. 1d,i)^35^. Importantly, only a small fraction of bulge SCs (up to four SCs per bulge) is genetically labelled by this particular approach allowing to trace individual bulge SCs^35^. Furthermore, SC marker CD34 and K15 are detected by immunofluorescence specifically within the HF bulge in young and older K14ΔNLef1 mice demonstrating that SC marker expression is still confined to bulge region in deformed HF structures (Supplementary Fig. 1d–g). As expected, 5 days after tumour initiation, YFP^+^ cells were detected within the bulge of deformed HF structures in epidermal whole mounts of K14ΔNLef1 mice (Fig. 1d). Subsequently, labelled cell clones expanded and an increase in the size of YFP^+^ cell clusters within epidermal whole mounts of early skin lesions were seen 2 weeks later (Fig. 1e,f). Four weeks following DMBA treatment, multiple small tumours were detected. Remarkably, the vast majority of tumours of TAM-treated mice contained a large number of YFP^+^ cells, whereas none were detected in oil-treated control mice (Fig. 1g–j). This was confirmed by analysing tumour sections (Supplementary Fig. 1h,i). Furthermore, progeny of bulge SCs were observed in tumours initiated in back and tail skin demonstrating that contribution of HF SCs to epidermal tumours was not specific to a particular body site (Fig. 1j). YFP^+^ cells were not only seen in the basal layer but were also found in the centre of tumour lobules co-expressing SCD1 demonstrating that differentiated tumour cells are derived from bulge SCs (Fig. 1h). Contribution of YFP^+^ cells to the entire tumour mass varied between 5 and 50% in whole mounts of tumours (Fig. 1g,h) suggesting that tumours are not monoclonal and are derived from more than one bulge SC. The detection of YFP^+^ and YFP^−^ cells in tumours could be due to the contribution of non-labelled bulge SCs or another cell population.

We and others have previously demonstrated that HF bulge cells contribute to the renewal of the SGs during skin homoeostasis^9^,^35^. Our results now show that bulge SCs also give rise to sebaceous tumours and constitute a cell-of-origin for well-differentiated epidermal tumours in the physiological environment and in the SC niche.

### Tumour-propagating cells identified in sebaceous tumours

**Figure 2:**
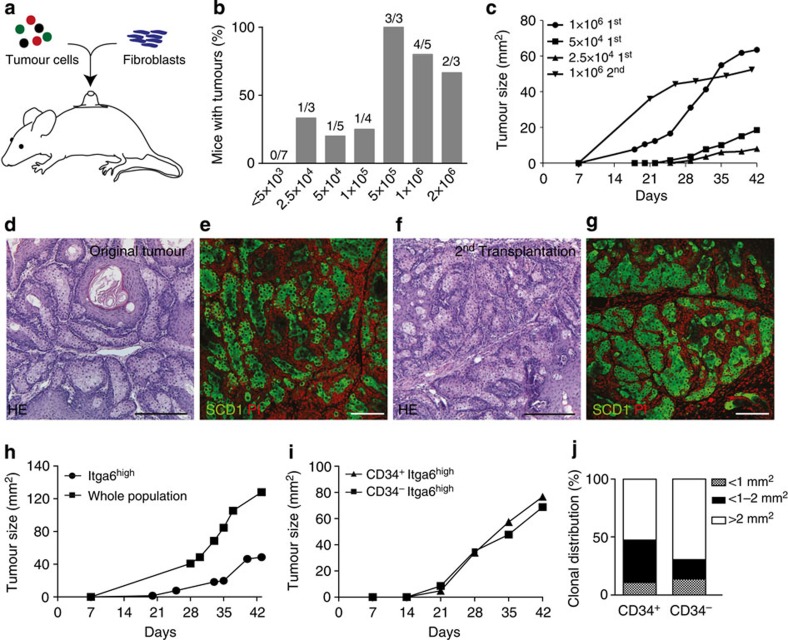
Identification of tumour-propagating cells in sebaceous tumours. (**a**) Transplantation assay of tumour cells into nude mice. (**b**) Engraftment of limiting dilutions of tumour cells. (**c**) Tumour size following transplantation of limiting dilutions and serial transplantations of sebaceous tumour cells. (**d**–**g**) hematoxylin and eosin (HE) (**d**,**f**) and immunofluorescent staining (**e**,**g**) for SCD1 (green) of original tumour of K14ΔNLef1 mice (**d**,**e**) and tumour developing following 2nd transplantation (**f**,**g**; *n*=6). Nuclei (red) stained with propidium iodide, (PI). (**h**,**i**) Tumour development and size in transplants of FAC-sorted Itga6^high^ cells (**h**) and CD34^+^/Itga6^high^ (**i**) compared with whole tumour cell population (**h**) and CD34^−^/Itga6^high^ fraction (**i**; Itga6^high^ *n*=6). (**j**) Colony-forming assay performed with CD34^+^/Itga6^high^ and CD34^−^/Itga6^high^ sorted tumour cells isolated from K14ΔNLef1 mice (*n*=3 mice, assay in triplicates). Scale bars, 50 μm.

We then went on to investigate whether well-differentiated sebaceous tumours contain cells with the ability to induce *de novo* tumours outside the normal SC niche (tumour-propagating cells). Therefore, we isolated primary tumour cells and performed transplantation assays into grafting chambers placed onto the back of nude mice (Fig. 2a)^37^,^38^. Surprisingly, serial dilution experiments revealed that ≥500,000 cells of a primary sebaceous adenoma generated robust tumour growth in recipient nude mice, demonstrating that the sebaceous adenomas were not hyperplastic SGs, but indeed contained transformed tumour cells with the ability to generate *de novo* tumours (Fig. 2b,c and Supplementary Fig. 2a). A total of 25,000 tumour cells were required for tumour formation following transplantation (Fig. 2b). The histology and architecture of the tumours generated by primary and secondary transplantation resembled the original sebaceous adenoma with prominent expression of the differentiation marker SCD1 within the tumour lobules (Fig. 2d–g). Sequential transplantation studies with keratinocytes isolated from secondary tumours demonstrated that a population of tumour-propagating cells was maintained to form sebaceous tumours (Fig. 2c). Furthermore, tumours persisted for a very long time after transplantation, demonstrating that tumour growth was not due to a transient response of tumour cells to the transplantation procedure but sustained stable growth for a long-time period.

The observation that well-differentiated sebaceous tumours contain tumour-propagating cells indicates that *de novo* tumour formation outside the physiological niche is not only a characteristic of malignant skin cancer, for example, SCC^2^,^23^,^39^.

To further explore the functional relevance of tissue SC characteristics for initiating tumour growth outside the natural SC niche, we set out to investigate whether tumour cells with a bulge SC surface signature (CD34^+^/Itga6^high^) possess a growth advantage following transplantation in a new physiological environment. Surprisingly, CD34^+^/Itga6^high^ and CD34^−^/Itga6^high^ tumour cells display similar tumour growth rates and sizes (Fig. 2i). Thus, selection of tumour cells for the expression of the bulge SC marker CD34 did not accelerate the tumour-initiating potential. Moreover, transplantation of whole tumour cell population leads to tumour growth 2 weeks earlier than Itga6^high^ basal tumour cells (Fig. 2h).

To further characterize the role of CD34^+^ tumour cells in propagating the tumour cell mass, the proliferative potential of sorted primary keratinocytes was assessed by performing clonogenicity assays, an established *in vitro* experiment to assess the proportion of SCs with high self-renewal and proliferative potential^40^. These experiments revealed that CD34^−^/Itga6^high^ tumour keratinocytes gave rise to larger cell clones (>2 mm^2^, holoclones)^40^, and therefore possessed a greater SC potential of self-renewal and higher proliferative capacity when compared with CD34^+^/Itga6^high^ tumour cells (Fig. 2j).

Our lineage tracing experiments demonstrated that bulge-derived SC progeny gave rise to sebaceous tumours. However, labelling of K15-positive pre-neoplastic lesions might exist and result in tumour growth. Therefore, we isolated and transplanted primary keratinocytes from young and aged K14ΔNLef1 mice and monitored growth of the transplants. Importantly, primary bulge and non-bulge keratinocytes did not result in tumour growth but led to the formation of deformed pilosebaceous units reflecting the phenotype of K14ΔNLef1 mice (Supplementary Fig. 2b,c).

Taken together, these experiments indicate that although bulge SCs are a cell-of-origin for sebaceous tumours in their normal SC environment, CD34^+^ tumour cells do not propel tumour cell propagation following transplantation. The data suggest that tumour initiation and tumour propagation are uncoupled and that distinct processes could potentially been driven by different cell populations. Our results imply either that Itga6^high^ undifferentiated/basal epidermal tumour cells cooperate with differentiated cells or that a hitherto unidentified cell population is capable to generate *de novo* sebaceous tumours following transplantation^23^.

### Targeting bulge SCs results in aggressive sebaceous tumours

**Figure 3:**
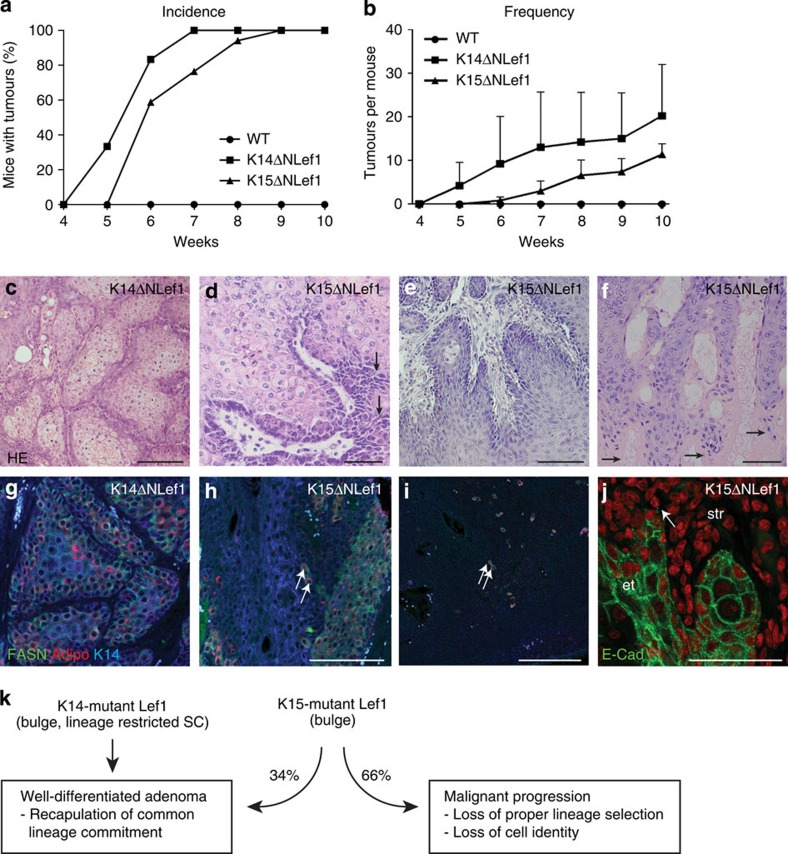
SC-driven mutant Lef1 leads to sebaceous tumour formation. (**a**,**b**) Incidence and frequency of tumours developing in K14ΔNLef1 (*n*=6), K15ΔNLef1 (*n*=17) and control (*n*=10) mice at indicated time points following DMBA application. Results are presented as mean±s.d.; *P*-values are K14ΔNLef1 versus wt 0.0209; K15ΔNLef1 versus wt 0.0062 and K14ΔNLef1 versus K15ΔNLef1 0.1738 (**b**). (**c**–**f**) Histology of representative tumours generated in K14ΔNLef1 (*n*=9) (**c**) and K15ΔNLef1 mice (*n*=58) (**d**–**f**). (**g**–**i**) Detection of K14 (blue) and sebocyte differentiation marker FASN (green) and Adipophilin (red) in tumour lobules of K14ΔNLef1 (*n*=3) (**g**) and K15ΔNLef1 (*n*=4 mice, 12 tumours) mice (**h**,**i**, arrows). (**j**) Immunofluorescent detection of E-Cadherin (green) reveals loss of cell adhesion in K15ΔNLef1 tumours and malignant invasion of tumour cells into surrounding stroma. PI (red) was utilized as nuclear counterstain. str, stroma; et, epithelial tumour (*n*=4 mice, 12 tumours). (**k**) Summary of tumour phenotypes seen in K14ΔNLef1 and K15ΔNLef1 mice. Scale bars, 50 μm.

To better understand the specific role of bulge SCs and their niche in driving tumour initiation and differentiation, transgenic mice expressing mutant Lef1 (ΔNLef1) under control of the bulge-specific K15 promoter were analysed (Supplementary Fig. 3e)^7^,^9^,^35^. Importantly and in contrast to K14ΔNLef1 mice, expression of mutant Lef1 was specific to the HF bulge region as demonstrated by immunofluorescent staining for the transgene (Supplementary Fig. 3a–f). Remarkably, K15ΔNLef1 mice developed spontaneous sebaceous skin tumours. Thus, we treated K14ΔNLef1 and K15ΔNLef1 mice with a single sub-threshold dose of the carcinogen DMBA to compare incidence and frequency of tumour formation (Fig. 3a,b). Nine weeks following DMBA treatment, all K14ΔNLef1 and K15ΔNLef1 mice had developed tumours with similar frequency (17.5±11.2 tumours/K14ΔNLef1 mouse and 18.1±6.88 tumours/K15ΔNLef1 mouse at 10 weeks following DMBA). As expected, DMBA treatment did not induce tumours in wild-type control mice (Fig. 3a,b). Tumours developing in both mouse lines were characterized by the presence of mature sebocytes (Fig. 3c,d), reflecting the central role of β-catenin/Lef1 signalling in governing keratinocyte lineage commitment^29^,^30^,^31^,^35^,^41^,^42^,^43^. Thus, inhibition of β-catenin/Lef1 activity in bulge SCs is sufficient to promote sebaceous tumour formation. These results also confirm our initial finding that bulge SCs constitute a cell-of-origin for sebaceous tumours.

Surprisingly, major differences were observed in the morphology of sebaceous tumours developing in K14ΔNLef1 and K15ΔNLef1 mice. Whereas benign sebaceous adenomas were observed in K14ΔNLef1 mice (Fig. 3c and Supplementary Fig. 1a), K15ΔNLef1 animals formed not only sebaceous adenomas but frequently also sebaceous tumours that were characterized by palisaded keratinocytes and spiky edges of abnormally formed tumour lobules (Fig. 3d,e). Moreover, sebaceous tumours of K15ΔNLef1 mice were more aggressive and often exhibited small and undifferentiated basaloid cells building an unorganized tumour mass with high mitotic activity (Fig. 3d–f). In addition to these aggressive sebaceous skin tumours, several tumours in K15ΔNLef1 mice were characterized by parakeratosis indicating a defect in differentiation of tumour cells (Fig. 3f, arrows).

### Cellular plasticity in SC-driven sebaceous tumours

The repertoire of sebaceous tumour types seen in SC-driven epidermal tumours was reflected by an impaired differentiation profile detected in the tumour tissues (Supplementary Fig. 4a). Marker molecules for mature sebocytes, FASN and Adipophilin, were not confined to the inner differentiated compartment of tumour lobules as seen in K14ΔNLef1 sebaceous adenomas (Fig. 3g). In contrast, only a few solitary marker-positive sebocytes were found within the more aggressive tumours generated in K15ΔNLef1 mice (Fig. 3h,i). Strikingly, keratinocytes of SC-driven tumours frequently invaded the surrounding stromal tissue as detected by the loss of expression of the membrane adhesion molecule E-cadherin confirming the undifferentiated and aggressive phenotype of K15ΔNLef1 tumours (Fig. 3j, arrows).

Our findings imply that sebaceous tumours differ with respect to the regulation and activity of the K14- and K15-promoter sequences driving the expression of mutant Lef1 in the different mouse models. Co-immunofluorescence staining revealed overlapping and distinct patterns of K14 and K15 expression in sebaceous tumours (Supplementary Fig. 4b–d). In addition, we compared expression levels of the transgene in HF bulge SCs isolated from K14ΔNLef1 and K15ΔNLef1 mice. Of note, the number of the bulge SCs was not significantly changed in K15ΔNLef1 mice when compared with K14ΔNLef1 mice (Supplementary Fig. 4f,g). Astonishingly, expression of mutant Lef1 was higher in CD34^+^/Itga6^high^ SCs from K14ΔNLef1 mice (Supplementary Fig. 4e) indicating that lower expression levels of mutant Lef1 driven by the K15 promoter allow for initiation of sebaceous tumours, which differ with respect to morphology, differentiation and aggressive growth.

Thus, our data show that the type of epidermal cell expressing mutant Lef1 impacts on tumour morphology and differentiation profile. These results underline that specific, cell-autonomous properties of distinct cell populations are not only crucial for generating a particular type of tumour but also for its grade of differentiation and malignant progression (Fig. 3k). Moreover, this important observation indicates that the level of oncogene expression by SCs is crucial for tumour initiation and highlights the specific and sensitive role of bulge SCs towards mutant Lef1 function.

**Figure 4:**
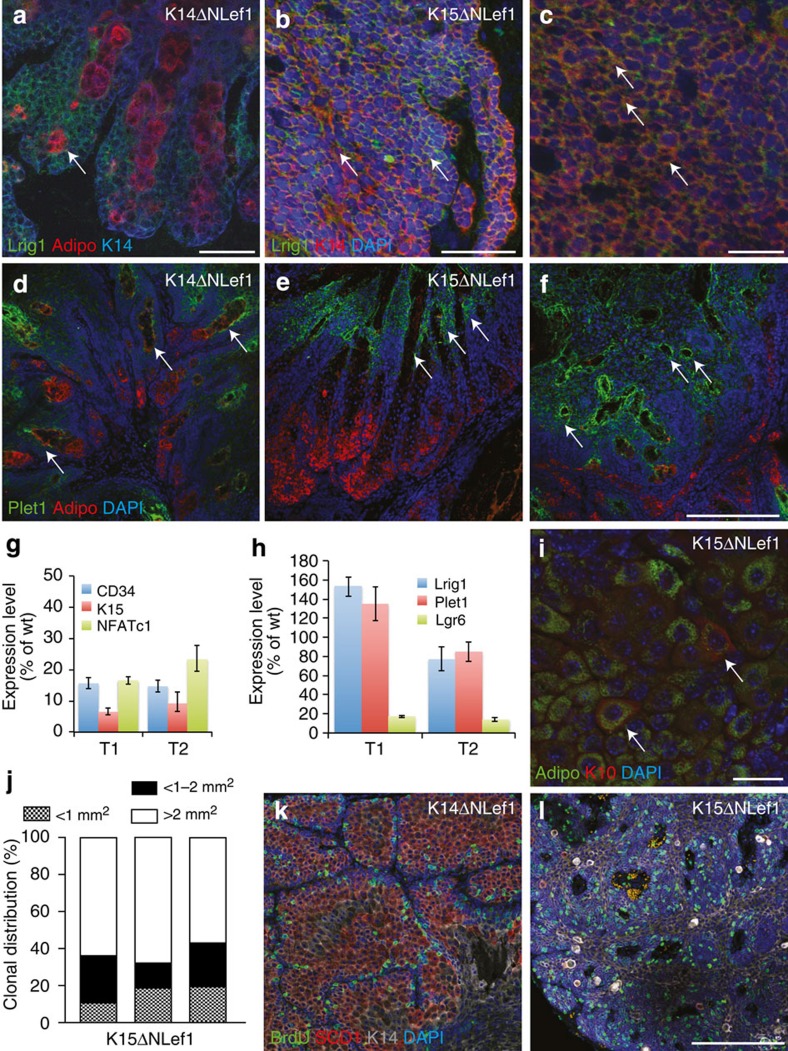
Cellular plasticity in SC-driven sebaceous tumours. (**a**–**c**) Immunofluorescent detection of Lrig1 (green), adipophilin (red), K14 (blue; **a**, arrows) and K14 (red) and nuclei (blue, 4',6-diamidino-2-phenylindole (DAPI)) (**b**,**c**) in tumours of K14ΔNLef1 (*n*=3) (**a**) and K15ΔNLef1 (**b**,**c**) mice (*n*=4 mice, 12 tumours). Note that K15ΔNLef1 tumours display Lrig1 expression in most of the cells (arrows in **b**,**c**). (**d**–**f**) Plet1 protein (green) localization in tumours isolated from K14ΔNLef1 (**d**) and K15ΔNLef1 (**e**,**f**) mice. Plet1 is confined to basal and ductal cells in differentiated tumours (**d**,**e**, arrows), whereas scattered throughout the tumour tissue (**f**) in the carcinoma. Adipophilin (red) depicts the mature sebocytes in tumour sections and nuclei (blue) are counterstained. (**g**,**h**) Expression of bulge SC marker CD34, K15 and NFATc1 (**g**) and JZ/I-marker Plet1, Lrig1 and Lgr6 (**h**) in tumours isolated from K15ΔNLef1 mice (T1 and T2) analysed by qRT–PCR. Samples were normalized to 18S and wild-type (wt) controls and s.d. was calculated (*n*=6 tumours, *n*=3 mice). (**i**) Defects in cell fate decision of tumour cells from K15ΔNLef1 mice as evidenced by Adipophilin (green)/K10 (red) staining in same cells (arrows; *n*=4 mice, 12 tumours). (**j**) Colony-forming assay with primary tumour cells isolated from K15ΔNLef1 mice (*n*=3). (**k**,**l**) BrdU (green) incorporation in tumours of K14ΔNLef1 (*n*=3) (**k**) and K15ΔNLef1 mice (*n*=4 mice, 12 tumours; **l**). Tumours are stained for SCD1 (red), K14 (grey) and nuclei (blue, DAPI) (*n*=6). Scale bars, 50 μm.

Different tumour phenotypes seen in K14ΔNLef1 and K15ΔNLef1 mice correlated with changes in SC and progenitor marker expression. HF bulge markers CD34, K15 and NFATc1 were decreased in tumours isolated from both K14ΔNLef1 and K15ΔNLef1 animals when compared with DMBA-treated skin of wild-type mice (Fig. 4g and Supplementary Fig. 5a). Expression of Lgr6, a SC marker for the IR, was also decreased in tumours isolated from both K14ΔNLef1 and K15ΔNLef1 animals (Fig. 4h and Supplementary Fig. 5a). In contrast, expression of Plet1 and Lrig1, markers for progenitors and SCs of the upper IR and JZ, respectively, were strongly expressed within SC-driven tumours (Fig. 4h). Robust expression of Lrig1 and Plet1 was also detected by immunofluorescence staining in K15ΔNLef1 tumours (Fig. 4b,c,e,f). Plet1 was predominantly detected in undifferentiated and adipophilin^−^ tumour cells localized to ductal structures facing the surface of the skin (Fig. 4d–f, arrows). In sebaceous adenoma of K14ΔNLef1 mice, Lrig1 was mainly confined to keratinocytes at the periphery of the tumour lobules (Fig. 4a, arrow). In contrast, aggressive sebaceous tumours of K15ΔNLef1 mice showed Lrig1^+^ cells distributed throughout the tumour mass (Fig. 4b,c). These data indicate that tumours generated in K15ΔNLef1 mice are endowed with an environment allowing propagation of more lineage-restricted SCs thereby leading to an enrichment of Lrig1^+^ SCs.

Remarkably, individual cells of SC-driven tumours co-expressed marker molecules for both SG (adipophilin/SCD1) and squamous differentiation (keratin 10; arrows Fig. 4i). This probably reflects a defect in controlling the lineage commitment and/or execution of differentiation programmes in K15ΔNLef1 tumour cells. Overlapping expression of lineage markers within a single tumour cell was never seen in sebaceous tumours developing in K14ΔNLef1 mice. This observation suggests that cellular plasticity in lineage commitment and loss of cell identity could result in the more aggressive phenotype seen in SC-derived tumours.

To test if proliferation is also altered in SC-driven tumours, we analysed 5-bromo-2′-deoxyuridine (BrdU) incorporation within tumour tissues. As expected, proliferating cells were confined to the undifferentiated basal keratinocytes at the periphery of tumour lobules in sebaceous adenoma of K14ΔNLef1 mice (Fig. 4k). In contrast, BrdU incorporation was detected throughout the tumour section of aggressive sebaceous tumours of K15ΔNLef1 mice (Fig. 4l) demonstrating that normal control of cell proliferation is lost in these SC-driven tumours. Previous reports showed that downregulation of Lrig1 correlated with a poor differentiation status and increased proliferation in human SCCs suggesting that loss of Lrig1 in SCC provides a growth advantage by upregulation of epidermal growth factor receptor signalling^44^,^45^. However, this correlation between Lrig1 expression and differentiation was not seen in SC-driven aggressive sebaceous tumours indicating that Lrig1 expression is differently regulated in different types of tumours.

To determine the proliferative potential of tumour cells, clonogenicity assays were performed with primary tumour cells isolated from K14ΔNLef1 and K15ΔNLef1 mice. Keratinocytes isolated from SC-driven tumours formed more large colonies (holoclones) and less small meroclones and abortive paraclones^40^ than primary tumour cells from K14ΔNLef1 animals (Fig. 4j and Supplementary Fig. 5b). Thus, SC-driven tumours are equipped with higher proliferative potential, which correlates with the development of more aggressive tumours.

### Abnormal p53 response in mutant Lef1 bulge SCs

**Figure 5:**
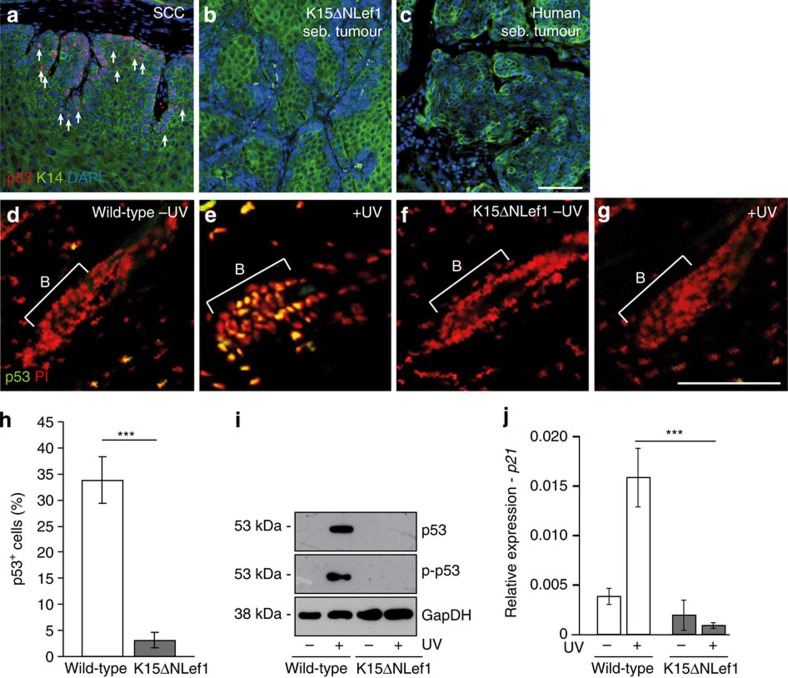
Defective p53 response in human sebaceous tumours and SCs of mutant Lef1 mice. (**a**–**c**) Immunofluorescent staining of p53 (red) and K14 (green) in SCC (*n*=2 mice, 3 tumours; **a**, arrows), sebaceous tumour of K15ΔNLef1 mice (*n*=4 mice, 10 tumours) (**b**) and human sebaceous tumour (**c**; *n*=7). (**d**–**g**) Immunofluorescent detection of p53 in HF bulge of wild-type (**d**,**e**) and K15ΔNLef1 mice (**f**,**g**) without (**d**,**f**) and following UV irradiation (**e**,**g**; *n*=3 mice). Nuclei stained in red (PI). (**h**) Quantification of p53 detection in HF of wild-type and K15ΔNLef1 mice following UV treatments (*n*=6 mice). Significance was calculated by Student’s *t*-test (***P<0.001) and s.d. was calculated. (**i**) Western blot analysis for p53, p-p53 and GapDH (loading control) in epidermal lysates from wild-type and K15ΔNLef1 epidermis 24 h following UV treatment (*n*=3 mice). (**j**) qRT–PCR analysis for *p21* mRNA expression in unexposed and UV-treated samples of wild-type and K15ΔNLef1 mice (*n*=6 mice). Significance was calculated by Student’s *t*-test (***P<0.001). Scale bars, 200 μm.

To identify the mechanism how mutant Lef1 initiates tumour growth in SCs, we first investigated one of the key players in cancer, p53, frequently mutated in SCC and BCC skin lesions. No p53 accumulation was detected in tumours generated in K15ΔNLef1 mice (Fig. 5b). Importantly, p53 was also not found in a panel of human sebaceous tumour samples (Fig. 5c). In contrast, the expected high number of p53^+^ tumour cells was seen in SCC control tumours as analysed by immunofluorescence stainings (Fig. 5a, arrows).

We then went on to perform ultraviolet (UV) irradiation experiments to examine whether normal p53 response is defective in HF bulge SCs expressing mutant Lef1. Generally, nuclear p53 accumulation is detected following UV irradiation of skin^46^,^47^ and accordingly, p53 was observed in HFs of control mice post ultraviolet treatment (Fig. 5d,e,h). This response was dramatically reduced in the HF bulge of K15ΔNLef1 mice (Fig. 5f–h). In addition, western blot experiments for p53 and p-p53 demonstrated that p53 activity was strongly impaired in UV-treated K15ΔNLef1 skin when compared with control tissue (Fig. 5i). Furthermore, expression of the p53 target gene *CDKN1A* was not induced following UV irradiation in epidermis of K15ΔNLef1 mice, whereas *CDKN1A* is highly expressed in UV-treated control epidermis (Fig. 5j).

Our data imply that normal p53 signalling is disturbed in SCs and tumours of K15ΔNLef1 mice. Thus, the p53 defect is an early event potentially contributing to SC-driven tumorigenesis as expression of mutant Lef1 results in a defective p53 response in HF bulge SCs.

### Defective SC proliferation and p53-independent apoptosis

Proper p53 function is crucial for the control of both apoptotic and cell cycle-regulating signals. Based on our finding that a normal p53 response is missing in HF SCs upon expression of mutant Lef1, we analysed these essential cellular functions in K15ΔNLef1 mice. To further explore the role of p53, we genetically deleted p53 from the entire epidermal tissue by crossing K15ΔNLef1 transgenic mice with p53^EKO^ mice (epidermal knock-out for p53; referred to as K15ΔNLef1/p53^EKO^ mice). p53^EKO^ mice were generated by crossing p53^fl/fl^ to K14Cre animals^48^,^49^. To verify that p53 was indeed lost from epidermis in K15ΔNLef1/p53^EKO^ and p53^EKO^ control mice, we analysed transcription levels of *p53* mRNA and nuclear accumulation of p53 protein following UV radiation. As expected, p53 protein was detected in numerous epidermal cells of wild-type and p53^fl/fl^ mice following UV treatment (Supplementary Fig. 6a). In contrast, no p53 signal was seen in p53^EKO^ keratinocytes (Supplementary Fig. 6b) demonstrating that p53 was efficiently deleted from mouse epidermis in p53^EKO^ mice. In addition, no or very little *p53* expression was detected by quantitative reverse transcription–PCR (qRT–PCR) in epidermis of p53^EKO^ mice, whereas normal *p53* transcript levels were seen in the epidermis of wild-type and K15ΔNLef1 mice (Supplementary Fig. 6c).

**Figure 6:**
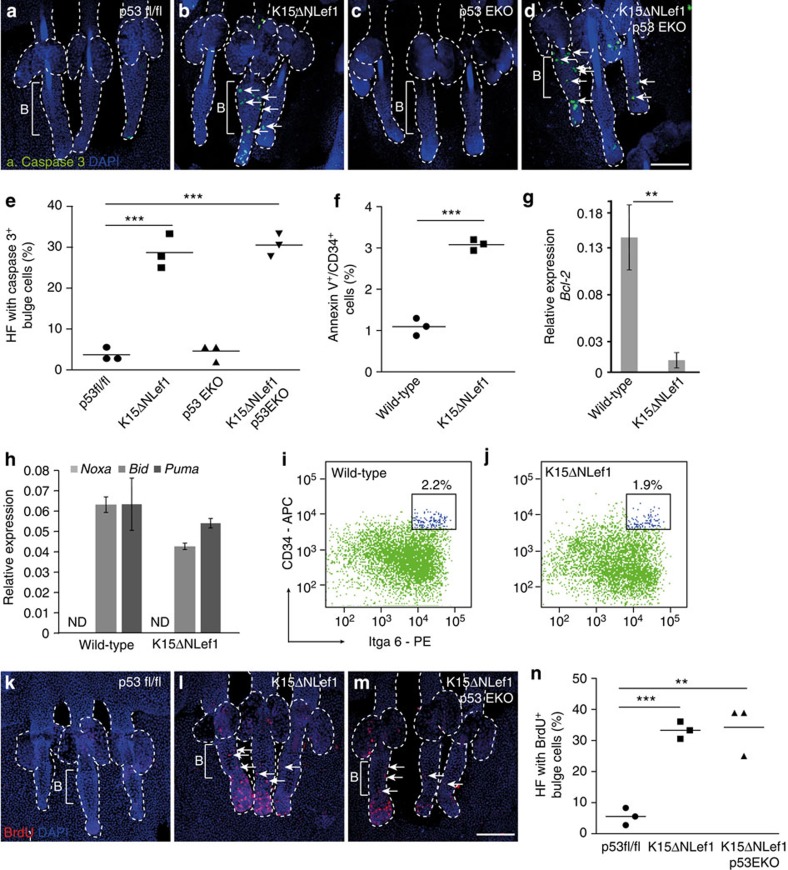
Defective SC homoeostasis preceding tumour formation. (**a**–**d**) Active caspase-3 detection (green, arrows) in epidermal whole mounts of p53fl/fl (**a**), K15ΔNLef1 (**b**), p53EKO (**c**) and K15ΔNLef1/p53EKO mice (**d**; *n*=3). (**e**) Quantification of HFs with caspase-3-positive cells within the HF bulge (*n*=3 mice; 40 HF per mouse). (**f**) FACS analysis of AnnexinV^+^ cells within the CD34^+^/Itga6^high^ SC compartment of wild-type and K15ΔNLef1 mice (*n*=3 mice). (**g**,**h**) qRT–PCR for *Bcl-2* (**g**) and *Noxa, Bid* and *Puma* mRNA expression (**h**) in CD34^+^/Itga6^high^ bulge SCs and CD34^−^/Itga6^high^ keratinocytes following UV treatment of skin of wild-type and K15ΔNLef1 mice (*n*=6 mice). ND, not detected. Samples were normalized to 18S and s.d. was calculated. (**i**,**j**) Quantification of CD34^+^/Itga6^high^ bulge SCs of wild-type (**i**) and K15ΔNLef1 (**j**) mice by FACS (*n*=4). (**k**–**m**) Analysis of BrdU incorporation (red, arrows) in epidermal whole mounts from p53fl/fl (**k**), K15ΔNLef1 (**l**) and K15ΔNLef1/p53EKO (**m**) mice (*n*=3). (**n**) Quantification of BrdU-positive cells in HF bulge (*n*=3 mice; 40 HF per mouse). Significance was calculated by Student’s *t*-test (**P<0.005, ***P<0.001). Scale bars, 400 μm. PE, phycoerythrin.

First, we investigated whether apoptosis is appropriately regulated in HF bulge SCs by analysing whole-mount epidermal tissue for cleavage of caspase-3 (CC3) by indirect immunofluorescent stainings. Typically, bulge SCs are quiescent in telogen, the resting phase of the hair cycle^50^ and do not undergo apoptosis. As expected, CC3 is not seen in HF of wild-type, p53^fl/fl^ and p53^EKO^ mice (Fig. 6a,c). Remarkably, CC3 was frequently detected within the HF bulge SC compartment of K15ΔNLef1 (Fig. 6b) and K15ΔNLef1/p53^EKO^ mice (Fig. 6d) demonstrating a significant increase in p53-independent apoptosis in bulge SCs upon expression of mutant Lef1 (Fig. 6e). In addition, the proportion of AnnexinV^+^ cells was significantly increased in CD34^+^/Itga6^high^ SCs isolated from K15ΔNLef1 mice when compared with wild-type littermates (Fig. 6f) further supporting our important observation that mutant Lef1 promotes apoptosis of SCs.

To further decipher the underlying mechanism of induced SC apoptosis in K15ΔNLef1 mice, we analysed the regulation of pro- and anti-apoptotic factors in bulge SCs. Previously, it has been shown that the anti-apoptotic factor Bcl-2 is strongly increased in bulge SCs upon genotoxic stress thereby preventing loss of SCs^51^. Interestingly, the increase in *Bcl-2* normally detected upon UV treatment of skin in bulge SCs of wild-type mice, was completely abolished in SCs of K15ΔNLef1 animals (Fig. 6g). In contrast, expression of pro-apoptotic molecules and p53 targets *BID* and *PUMA* were not differently regulated in SCs of K15ΔNLef1 skin when compared with wild-type littermates (Fig. 6h). Of note, the anti-apoptotic factor *NOXA* was not detected in bulge SCs (Fig. 6h). Thus, K15ΔNLef1 mice have a defect in SC-specific regulation of Bcl-2 thereby allowing apoptosis of HF bulge SCs.

Despite the strong induction of apoptosis of normally quiescent bulge SCs, K15ΔNLef1 mice do not exhibit obvious defects in HF renewal during adulthood. Indeed, analysis of the bulge SC compartment revealed that the number of HF SCs was not significantly changed in K15ΔNLef1 mice when compared with wild-type littermates (Fig. 6i,j). Therefore, we wondered if proliferation is altered in HF of K15ΔNLef1 mice and tested epidermal whole mounts of K15ΔNLef1 and control mice for BrdU incorporation. Typically, the rate of cell division is very low in SCs of telogen HF (Fig. 6k). However, a strong increase in the number of BrdU^+^ cells was detected in whole mounts of K15ΔNLef1 mice (Fig. 6l,n). This significant boost in proliferation was also observed in K15ΔNLef1/p53^EKO^ mice (Fig. 6m,n). Thus, our data demonstrate that mutant Lef1 induces apoptosis of bulge SCs and that this is compensated by the expansion of HF keratinocyte to maintain integrity of the pilosebaceous unit. Hence, mutant Lef1 balances both cellular functions in a p53-independent manner in SCs.

### Abnormal DNA damage response by mutant Lef1 SCs

**Figure 7:**
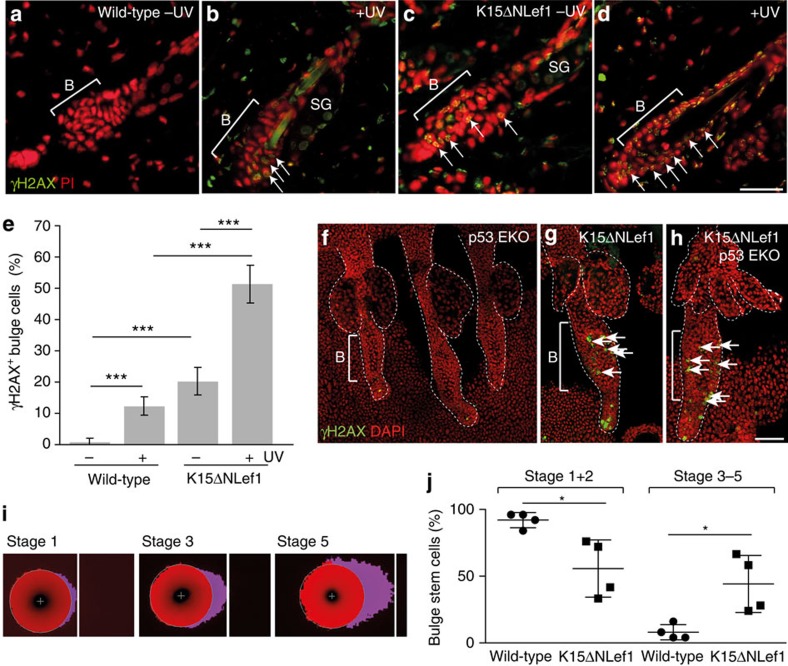
Mutant Lef1 induces DNA damage in bulge SCs. (**a**–**d**) γH2AX foci (arrows) in wild-type (**a**,**b**) and K15ΔNLef1 (**c**,**d**) mice without (**a**,**c**) and following UV irradiation (**b**,**d**). (**e**) γH2AX foci quantification following UV irradiation (*n*=4 mice). Significance was calculated by Student’s *t*-test (***P<0.001) and s.d. was calculated. (**f**–**h**) γH2AX foci (arrows) in epidermal whole mounts of p53^EKO^ (**f**), K15ΔNLef1 (**g**) and K15ΔNLef1/p53^EKO^ (**h**) mice (*n*=3). (**i**,**j**) Representative images of Stage 1, Stage 3 and Stage 5 comets of CD34^+^/Itga6^high^ bulge SCs (**i**) and quantification of four independent assays (*n*=25) on bulge SCs from wild-type and K15ΔNLef1 mice (**j**). Significance was calculated by Student’s *t*-test (*P<0.05). Scale bars, 85 μm (**a**–**d**), 250 μm (**f**–**h**). DAPI, 4',6-diamidino-2-phenylindole.

Owing to the fact that normal p53 response constitutes an essential surveillance mechanism triggering accelerated DNA repair activity and given our observation that apoptosis of SCs is induced upon expression of mutant Lef1, we next sought to delineate how SCs initiate tumour formation in K15ΔNLef1 mice. In a first set of experiments, we analysed bulge SCs of K15ΔNLef1 transgenic mice for alterations in DNA damage repair. Astonishingly, we identified γH2AX foci within the HF bulge of K15ΔNLef1 mice, which are indicative for on-going DNA damage (Fig. 7c,e,g). In contrast, HF of littermate control mice did not display this γH2AX signal (Fig. 7a,e,f). Next, we performed UV-B treatment of mice leading to DNA damage in keratinocytes and the detection of γH2AX foci in wild-type mice (Fig. 7b,e). However, UV treatment of K15ΔNLef1 mice propelled an even stronger DNA damage and γH2AX foci were seen in 50% of bulge SCs (Fig. 7d,e). Importantly, K15ΔNLef1/p53^EKO^ mice exhibited a high number of γH2AX foci in HF SCs without UV treatment (Fig. 7h) demonstrating that the p53 defect was not the driving force of DNA damage in mutant Lef1 SCs.

To test whether DNA is indeed damaged in SCs of K15ΔNLef1 mice, we performed comet assays to detect DNA double strand breaks (DSBs)^52^. In this assay, DNA containing DSBs is visualized in comet tails and the relative tail intensity correlates with the level of DNA DSBs^53^. In particular, tails with smaller intensity (Stage1 and 2) possessed very low levels of DNA damage, whereas comet tails with high intensities (Stage 3–5) reflected fragmented DNA and hence high levels of DNA DSBs (Fig. 7i). Quantification of four independent experiments revealed that bulge SCs isolated from K15ΔNLef1 mice showed lower number of Stage1 and 2 comets when compared with SCs from wild-type littermates. In contrast, comets of Stage 3–5 were significantly increased in bulge SCs form K15ΔNLef1 mice (Fig. 7j). Thus, our data demonstrate for the first time that expression of mutated transcription factor Lef1 leads to the accumulation of DNA damage in epidermal SCs.

**Figure 8:**
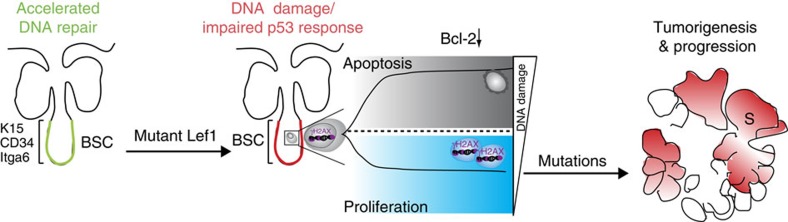
Model for SC-driven epidermal tumour initiation by mutant Lef1. Expression of mutant Lef1 results in defective DNA damage response and impairment of SC-specific surveillance mechanisms, including p53 activation. SCs γH2AX carrying high levels of DNA damage undergo apoptosis, mainly by blocking the normal Bcl-2 response. Bulge SCs (BSCs) with lower degree of damaged DNA escape normal control of SC proliferation to maintain the epidermal tissue. Consequently, these proliferative SCs carrying DNA breaks accumulate further mutations, thereby initiating tumour formation.

In conclusion, our study identifies a novel mechanism for SC-driven tumour initiation *in vivo*. It was previously shown that bulge SCs are characterized by accelerated DNA repair activity and concomitant attenuation of p53 activation^51^. This mechanism for protecting SCs against DNA damage-induced cell death is impaired upon expression of mutant Lef1. Our data demonstrate that damaged DNA is not recognized by the p53-checkpoint axis and consequently, DNA lesions are not efficiently repaired in these mice. Thus, SCs carrying damaged DNA escape normal control of proliferation allowing further expansion and subsequent tumour formation (Fig. 8).

## Discussion

In this study, we identify novel, divergent functions of epidermal SCs in the process of tumour initiation and the control of the tumour phenotype and tumour cell identity. An inducible *in vivo* model for sebaceous skin tumours was used as one example for well-accessible solid tumours with a known differentiation profile and localization of marker molecules. Given that diverse pools of stem and progenitor cells have been characterized within certain regions of the HF in mammalian skin^5^, our tumour model thus allows to address important questions on the function of different SCs in tumorigenesis as well as investigating the underlying SC-specific molecular mechanisms. Better understanding these mechanisms will be beneficial to develop effective therapeutic strategies to specifically target the relevant cell population initiating and steering tumour growth.

Lineage tracing experiments of bulge SCs within an inducible skin tumour model for sebaceous adenomas show clonal expansion of individual labelled SCs and contribution of labelled cells to tumour formation in high frequency. Thus, bulge SCs serve as cellular source for well-differentiated epidermal tumours in their natural niche. Based on our observation that sebaceous tumours are not monoclonal derived, other un-labelled bulge SCs or cells outside the bulge compartment could potentially contribute to tumour growth. Thus, our study sheds light on the important fact that the process of tumour initiation impacts on tumour heterogeneity often seen in epidermal cancer.

By transplantation experiments, we identified tumour-propagating cells in well-differentiated epidermal tumours. In this experimental setup, bulge SC signature does not result in a tumour growth advantage. Our experiments imply that although bulge SCs are a cell-of-origin for differentiated epidermal tumours in their normal SC environment, tumour cell propagation and tumour initiation are not enhanced following transplantation of tumour cells with bulge SC signature. In addition, expression of bulge SC marker molecules, including NFATc1, K15 and CD34 were decreased in sebaceous tumours. In contrast, a pool of more lineage-restricted SCs, for example, Lrig1^+^ tumour cells, is enriched and therefore, could drive tumour growth outside the normal SC niche. Alternatively, transplantation could select for tumour cells that dedifferentiate as it has elegantly been shown for intestinal tumorigenesis. In this experimental setting, tumour initiation involves a bidirectional conversion of differentiated cells into SC-like populations therefore causing cellular plasticity^54^. It is tempting to speculate that those mechanisms are also applied during skin tumorigenesis, but this certainly awaits further investigations.

Based on the observation that mice expressing mutant Lef1 in different epidermal compartments generate a diverse repertoire of sebaceous tumours, we conclude that the cell population that carries the genetic mutations and thus drives tumour formation has a major impact on the tumour phenotype and grade of malignancy. SC-driven tumours exhibit a heterogeneous and aggressive phenotype. The differentiation defects observed in K15ΔNLef1 mice might be a consequence of an erroneous response towards instructive signals and could promote aggressive growth of the affected tumour cells. This observation suggests that cellular plasticity in lineage commitment and loss of cell identity result in dedifferentiation of tumour cells and could be part of the malignant progression of SC-driven tumours. In addition, expression levels of mutant Lef1 were different in SCs isolated from K14ΔNLef1 and K15ΔNLef1 mice indicating a specific and sensitive response of bulge SCs towards abnormal Lef1 activity. The results suggest that the expression level of any oncogene by SCs significantly impacts on the process of tumour initiation.

Human sebaceous lesions are often associated with hereditary colorectal carcinomas and the Torre-Muir syndrome that involve a loss of DNA mismatch repair proteins MLH1 and MSH2 (ref. 28). Interestingly, overexpression of mutant Lef1 in bulge SCs leads to an impaired DNA damage response, independent of impaired p53 function. Generally, bulge SCs are protected from accumulation of mutations by a transient and fast p53 stabilization and fast DNA repair activity^51^. As summarized in the model presented in Fig. 8, mutant Lef1 signalling impairs these SC-specific surveillance mechanisms and DNA DSB lesions are detected in bulge SCs of K15ΔNLef1 mice. As a consequence, SCs carrying particular high levels of DNA damage undergo apoptosis. Mechanistically, our data identify an important role of mutant Lef1 in blocking the normal anti-apoptotic Bcl-2 response seen in bulge SCs following genotoxic stress^51^ (Fig. 8). To maintain tissue integrity, SCs with lower degree of damaged DNA escape normal control of cell division. Indeed, numerous proliferative bulge SCs are detected in mutant Lef1 mice. These results suggest that proliferating SCs carrying DNA breaks accumulate further mutations driving the process of tumour initiation and propagation (Fig. 8). In the future, the type of genetic aberrations involved in sebaceous tumour growth requires further characterization. Here we have identified a SC-specific gatekeeper mechanism protecting against tumour initiation in mammalian skin and additional studies will unravel if similar defects in SC surveillance functions contribute to tumorigenesis in other tissues. In particular, our data could also have implications for other tissues for which TCF/Lef1 signalling has been shown to play a pivotal role in governing SC activation and proliferation.

## Methods

### Experimental mice

K15CreER(G)^T2^, R26REYFP, p53^fl/fl^, K14Cre, K14ΔNLef1 and K15ΔNLef1 mice have been described before^31^,^35^,^36^,^48^,^49^.

K15CreER(G)^T2^ mice were crossed with R26REYFP mice. Cre reporter lines were always kept in a homozygous state to maximize visualization of positive recombinants. To perform lineage tracing experiments in the process of tumour formation, K15CreER(G)^T2^/R26REYFP (K15_YFP) were crossed into the K14ΔNLef1 background (K15_YFP/K14ΔNLef1). K15ΔNLef1 mice were crossed to K14Cre and p53^fl/fl^ mice to generate K15ΔNLef1/p53^EKO^ mice. All mouse strains were maintained on a C57Bl/6 background. All *in vivo* experiments were carried out according to guidelines and an animal license given by the State Office North-Rhine Westfalia.

### Tumour experiments

To initiate tumorigenesis, 7-week-old K14ΔNLef1 (*n*=6), K15ΔNLef1 (*n*=17), wild-type (*n*=10) and K15_YFP/K14ΔNLef1 mice (*n*=6) were treated once with a sub-threshold dose of DMBA (150 nM, Sigma-Aldrich)^30^. Three to four weeks after topical DMBA application tumour formation was observed. Tumours were monitored and scored once per week.

### Irradiation experiments

Mice were treated with one single dose of ultraviolet-B (0.36 J m^−2^) using a Waldmann TP-4 lamp. Unexposed and exposed skin were harvested either 4 or 24 h after irradiation.

Tissue samples were fixed in 4% paraformaldehyde and possessed for paraffin sectioning and subsequently stained with indicated antibodies. For western blot analysis (Supplementary Fig. 7), whole epidermal proteins were isolated using Trizol reagent (Invitrogen), according to the manufacturer’s instructions. A measure of 15 μg protein was loaded on 12% Tris-Glycine gel. Proteins were then transferred to polyvinylidene difluoride membrane. Membranes were probed with antibodies against p53 (1:2,000, Cell Signaling), p-p53 (1:2,000, Cell Signaling) and GapDH (1:2,000, Abcam) followed by incubation with anti-mouse or anti-rabbit secondary antibodies conjugated with horseradish peroxidase (1:5,000, GE Healthcare). Specific protein bands were visualized with an ECL Prime Western Blotting Detection Reagent (GE Healthcare). Western blot experiments were repeated twice.

### Lineage tracing during epidermal tumorigenesis

To investigate a potential contribution of K15-derived progeny to tumour formation, Cre recombinase in K15_YFP/ K14ΔNLef1 was induced by TAM at 3 consecutive days prior or following DMBA treatment. TAM was applied intraperitoneally with a dose of 2.5 mg per day.

Mice were killed and tumour tissue was harvested and fixed in 4% formaldehyde or 0.2% glutaraldehyde/2% formaldehyde for paraffin sections. Alternatively, tissue was equilibrated in 30% sucrose over night and embedded in tissue Tec OCT (Sakakura). For YFP detection, tumours were fixed with 4% paraformaldehyde before incubation with 30% sucrose over night at 4 °C.

Mice were injected with 100 mg kg^−1^ bodyweight BrdU (Sigma-Aldrich). Tumour tissue was harvested 1.5 h post injection, fixed in 4% formaldehyde and embedded in paraffin. BrdU incorporation was detected by immunofluorescent staining.

### Isolation of epidermal whole mounts

Epidermal whole mounts of tail skin were isolated as described before^55^,^56^. To analyse epidermal whole mounts of early lesions and tumours, tissue was dissected and fat and connective tissue was removed. Following digestion with 20 mM EDTA, epithelium was gently peeled off using forceps. Subsequently, whole mounts were fixed in 3.4% formaldehyde or 0.2% glutaraldehyde/2% formaldehyde for 2 h at room temperature.

### Immunofluorescence staining

Epidermal whole mounts and sections were stained according to the published protocols^35^,^55^. Following primary antibodies were used: K15 (mouse, Neomarkers, 1:1,500), K14 (rabbit, Covance, 1:3,000), K10 (rabbit, Covance, 1:500), BrdU (mouse, BD Bioscience, 1:50), BrdU (rat, Oxford Biotechnologie, 1:500), adipophilin (guinea pig, Fitzgerald Industries, 1:1,500), active Caspase-3 (rabbit, R&D Systems, 1:500), SCD1 (goat, Santa Cruz, 1:150 and rat, R&D Systems, 1:150), Lrig1 (goat, R&D Systems, 1:100), Plet1 (rat, MUbio, 1:500), E-cadherin (mouse, BD Bioscience, 1:1,000), FASN (G-11) (mouse, Santa Cruz, 1:100), Lef1 (rabbit, Cell Signaling, 1:100), γH2AX (rabbit, Cell Signaling, 1:200), p53 (rabbit, Novocastra/Leica, 1:500), p53 (mouse, Cell Signaling, 1:100) and 9E10 (rabbit, Santa Cruz, 1:200). All secondary antibodies coupled to Alexa-488, Alexa-594, Cy-5 or Cy-3 were obtained from Molecular Probes and GFP antibody was coupled to Alexa-488 (goat, Rockland, 1:250). Immunostainings were performed twice.

### Fluorescence-activated cell sorting (FACS)

Skin and tumour samples were harvested, minced with a scalpel and incubated for 45 min at 37 °C in 0.25 mg ml^−1^ Thermolysin (Sigma) in DMEM/HAM’s F12 low-calcium medium (Biochrom). The suspension was filtered and washed with DMEM/HAM’s F12 low-calcium medium. Afterwards, cells were suspended either in DMEM/HAM’s F12 low-calcium medium supplemented with 100 U ml^−1^ penicillin, 100 μg ml^−1^ streptomycin, 1.8 × 10^−4^ M adenine, 2 mM L-glutamine, 0.5 μg ml^−1^ hydrocortisone, 10 ng ml^−1^ EGF, 10^−5^ M cholera enterotoxin, 5 μg ml^−1^ insulin and 10% FCS Gold for subsequent engraftment experiments or in 5% FCS/PBS for FACS experiments. For FACS staining, cell suspensions were incubated in 5% FCS/PBS for 30 min at 4 °C with the following antibodies: phycoerythrin-conjugated α6-integrin (CD47f, BD Pharmingen; 1:25), AlexaFluor647-coupled CD34 (Ram34, eBioscience; 1:10). To analyse apoptosis (FITC)-conjugated Annexin V (kind gift from B.Brachvogel, 1:60) was added. Cell viability was assessed by 7AAD (BD Bioscience) labelling. Subsequent analysis was carried out using a FACSCantoII Cytometer (BD Bioscience) equipped with BD FACSDiva Software. Cell sorting (α6-integrin^high^/CD34^+^ versus α6-integrin^high^/CD34^−^) on a FACSAriaIII cell sorter (BD Bioscience) was performed to specifically analyse the bulge SC compartment in engraftment studies and colony-forming assay.

### Tumour cell transplantation

Grafting studies were performed on the back skin of anaesthetized Balb/c-nude mouse (Charles River) as described before 37,38. Briefly, a silicon chamber (Renner GmbH) was implanted into a small excision wound. Each grafts consisted of freshly isolated or sorted tumour cells (5 × 10^3^–2 × 10^6^) mixed with 2 × 10^6^ primary dermal fibroblasts from newborn mice. In some experiments, primary keratinocytes were grafted together with fibroblasts. A volume of 50 μl cell suspension was injected into the implanted silicon chamber. After 1 week, chambers were removed and 2 weeks following transplantation, tumour growth was monitored twice a week.

### Colony-forming assay

Triplicates of 2,500 and 5,000 freshly isolated tumour cells derived from K14ΔNLef1 or K15ΔNLef1 mice (*n*=3) were plated onto collagen G-coated six-well plates (0.04 mg ml^−1^, Biocoat, BD Biosciences). Cells were co-cultured with mitomycin-treated J23T3 feeder cells (Swiss Albino) in defined low-calcium keratinocyte DMEM/HAM’sF12 medium^40^,^57^. In a different set of experiments, tumour cells (*n*=3 tumours per genotype) were separated into CD34^+^/Itga6^high^ and CD34^−^/Itga6^high^ fractions by FACS. Subsequently, 3,000 and 6,000 sorted cells were seeded. Clonal growth was assessed for 2–4 weeks of cultivation. Cells were fixed with 4% paraformaldehyde and stained with rhodanil (1% rhodamine/2% nileblue) 40. Colony-forming efficiency and clone size were evaluated using ImageJ (NIH) software and calculated as number of colonies/plated cells in %.

### qRT–PCR analysis

Isolation of total RNA from epidermis, FAC-sorted bulge SCs and skin tumours and quantitative real-time PCR experiments for expression of bulge SC marker and lineage-restricted SC marker molecules were performed^35^. In addition, the following primers were used:

Bcl-2

5′- ATGGGGTGAACTGGGGGAGGATTG -3′ (forward)

5′- GGCCAGGCTGAGCAGGGTCTTC -3′ (reverse)

Bid

5′- AACCGCGACCATGGAAAGACC -3′ (forward)

5′- ATGCAGGAGCCGGCGTAAACT -3′ (reverse)

Noxa

5′- CGCTTGCTTTTGGTTCCCTGAG -3′ (forward)

5′- CAAACGACTGCCCCCATACAAT -3′ (reverse)

Myc-Tag

5′- GATCAGCGAGGAGGAC -3′ (forward)

5′- CTAAGTCGCCCTCCTCTTC -3′ (reverse)

Puma

5′- CGGCGGAGACAAGAAGAG -3′ (forward)

5′- GAGGAGTCCCATGAAGAGATTG -3′ (reverse)

p21

5′- CTGAGCGGCCTGAAGATT -3′ (forward)

5′- GCTAAGGCCGAAGATGGGGAAGA -3′ (reverse)

p53

5′- TCATCTTTTGTCCCTTCTC -3′ (forward)

5′- TTATTGAGGGGAGGAGAG -3′ (reverse)

Standard derivations are presented for technical replicates. Three independent experiments were performed and analysed.

### Comet assay

FAC-sorted α6-integrin^high^/CD34^+^ cells were used in neutral comet assay^52^.

### Statistical analysis

Statistical significance was calculated using Student’s *t*-test, except for analysing differences in tumour frequency. Statistical analysis of differences in tumour frequency was carried out using two-way analysis of variance with repeated measures. Quantification methods are indicated in the respective figure legends. All *P*-values below 0.05 were considered significant. Statistical analyses were done using Microsoft Excel and GraphPad Prism.

## Author contributions

The study was designed by C.N. and M.P. Tumour experiments and analysis were performed by C.N., M.P. and A.K. Lineage tracing of SCs was done by M.P. Immunostainings were performed by K.R., M.P. and H.B. Clonogenicity assay was performed by M.P and P.S. and transplantations by H.B., A.K. and M.P. FACS analysis was done by H.B., M.P., K.R. and P.S., M.P. performed qRT–PCR studies. K.R. did western blotting, ultraviolet treatment and comet experiments. C.N. and M.P. wrote the manuscript. All authors discussed the results and commented on the manuscript.

## Additional information

**How to cite this article**: Petersson, M. *et al*. Interfering with stem cell-specific gatekeeper functions controls tumour initiation and malignant progression of skin tumours. *Nat. Commun.* 6:5874 doi: 10.1038/ncomms6874 (2015).

## Supplementary Material

Supplementary Information Supplementary Figures 1-7
