# Supplementary material for: Interfering with stem cell-specific gatekeeper functions controls tumour initiation and malignant progression of skin tumours
Source: Nat Commun. 2015 Jan 22;6:5874. doi: 10.1038/ncomms6874 (PMC4354047; doi:10.1038/ncomms6874)
Supplement: Supplementary Information — Supplementary Figures 1-7 [file ncomms6874-s1.pdf]

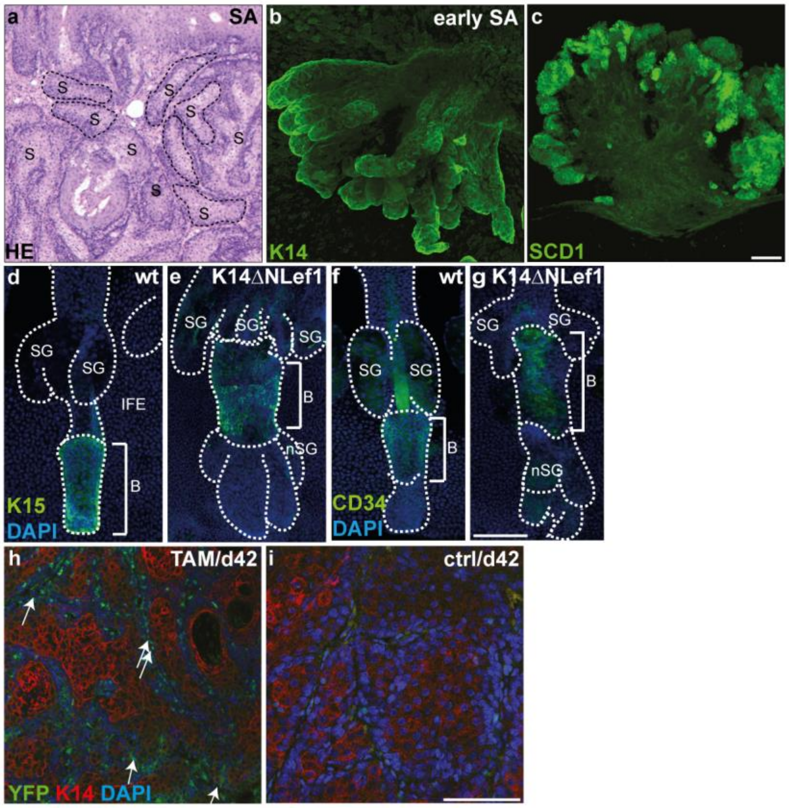

Supplementary Figure 1. Sebaceous tumour phenotypes developing in K14ΔNLef1 mice.

(a) H&E stain of tumour sections from early sebaceous lesion.
(b,c) Immunofluorescent stainings of K14 (green) (**b**) and SCD1 (green) (**c**) in whole mounts of sebaceous tumours. **(d-g)** Detection of bulge stem cell marker K15 (green) (**d,e**) and CD34 (green) (**f,g**) in aged control (wt) and K14ΔNLef1 mice. Counterstain with DAPI (blue) (n=3). Scale bar 200 µm. SA, sebaceous adenoma; B, bulge; IFE, interfollicular epidermis; nSG, *de novo* sebaceous gland, SG, sebaceous gland. **(h,i)** Detection of EYFP (green), K14 (red) and nuclei (blue) in tumours of K14CreER(G)$_{T2}$/K14ΔNLef1/R26YFP mice 42 days following application of TAM (**h**) or oil (**i**).Scale bar 50µm.

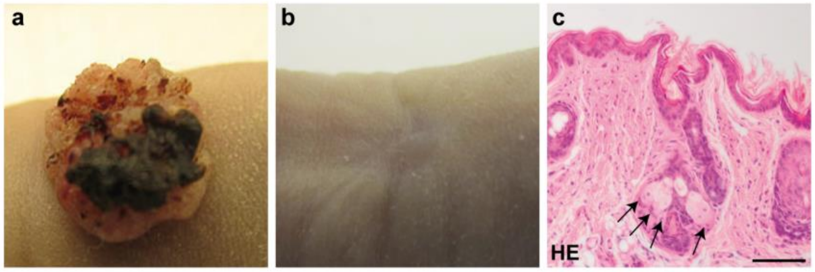

Supplementary Figure 2. Transplantation of sebaceous tumour cells and mutant Lef1 keratinocytes.

(a) Tumour derived from transplanted sebaceous tumour cells ($1x10^6$). **(c,b)** Skin of nude mice transplanted with sorted Itga6^{high}/CD34^{+ve} isolated from aged K14ΔNLef1 mice. Note, no tumours develop following transplantation, but deformed hair follicle and multiple sebaceous glands are generated reflecting the phenotype of K14ΔNLef1 mice (arrows in c). Scale bar 200μm.

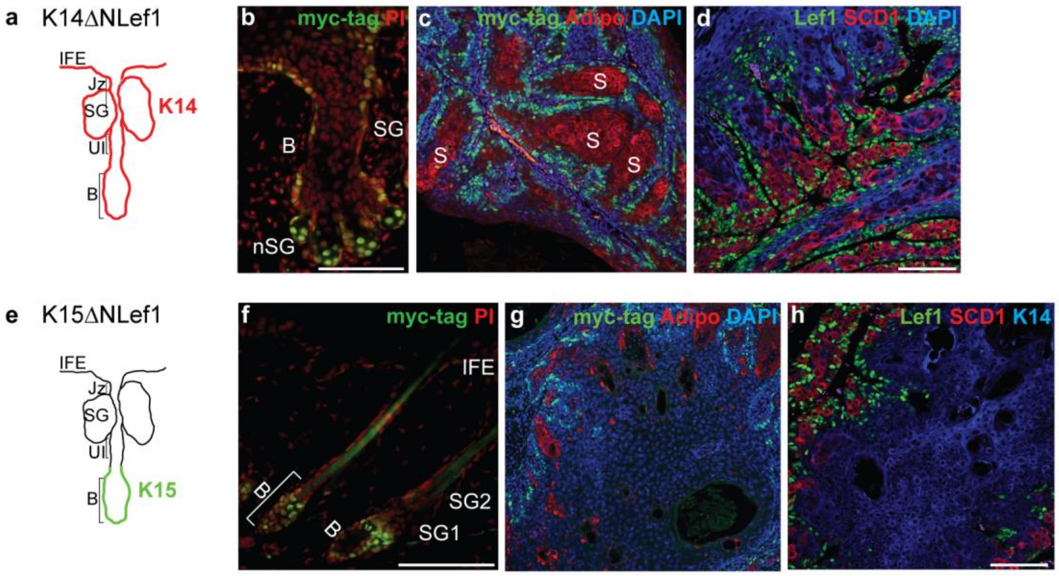

Supplementary Figure 3. Characterisation of skin phenotype of K14ΔNLef1 and K15ΔNLef1 transgenic mice.

(a-d) Generation of K14ΔNLef1 mice and analysis of transgene expression in skin (n=5 mice) (myc-tag, green) (**b**) and in sebaceous adenomas (myc-tag, green) (**c**) (Lef1, green) (**d**). Immunofluorescent staining for sebocyte marker adipophilin and SCD1 (both in red) demonstrate sebocyte differentiation of tumours (n=3). B, bulge; IFE, interfollicular epidermis; Jz, junctional zone; nSG, new additional sebaceous gland; S, sebaceous tumour lobules; SG, sebaceous gland; UI, upper isthmus. **(e-h)** Generation of K15ΔNLef1 mice and analysis of transgene expression in skin (n=5 mice) (myc-tag, green) (**f**) and in sebaceous tumours (myc-tag, green) (**g**) (Lef1, green) (**h**). Immunofluorescent staining for sebocyte marker Adipophilin and SCD1 (both in red) demonstrates reduced sebocyte differentiation of tumours (n=4 mice, 12 tumours). Scale bars 50µm.

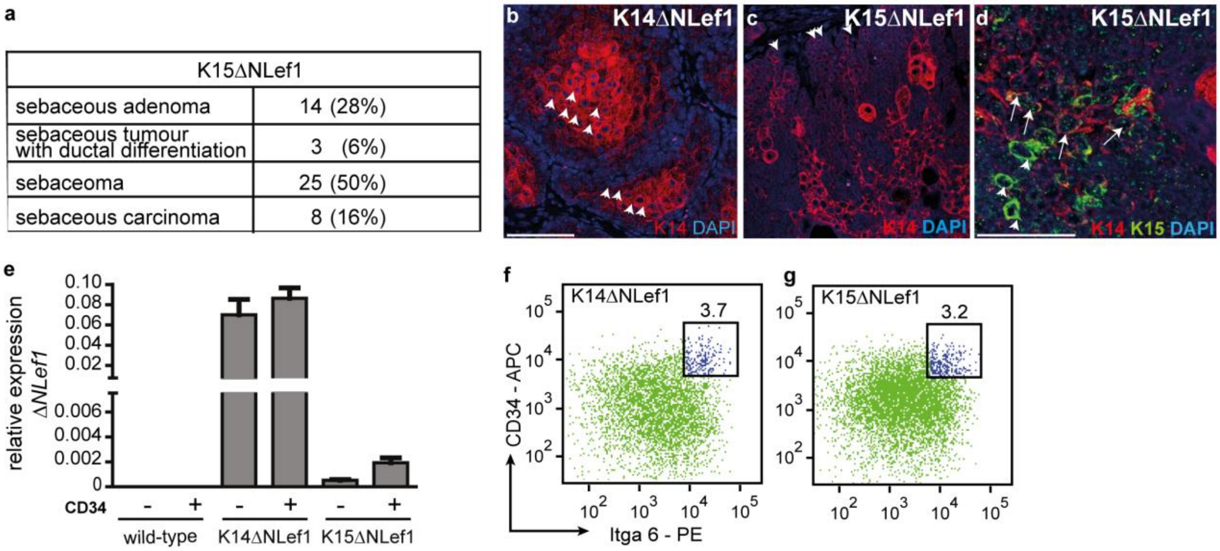

Supplementary Figure 4. Tumour classification and transgene expression in K14ΔNLef1 and K15ΔNLef1 transgenic mice.

(a) Classification of DMBA-induced skin tumours from K15ΔNLef1 (n=9 mice, 31 tumours). (**b-d**) Immnunofluorescent staining for K14 (red) (**b-d**) and K15 (red) (**d**) in tumours from K14ΔNLef1 (**b**) and K15ΔNLef1 mice (**c,d**). Note low K14 protein in basal K15ΔNLef1 tumour cells (arrow head in **c**) compared to K14ΔNLef1 (arrow head in b) and detection of K15-positive (arrow heads) and K14/K15-double positive cells in sebaceous tumours (arrows in **d**). (**e**) Analysis of transgene expression by qRT-PCR in Itga6^{high}/CD34^{-ve} and Itga6^{high}/CD34^{+ve} FAC-sorted cells isolated from wild-type, K14ΔNLef1 and K15ΔNLef1 mice. Samples were normalized to 18S and SD was calculated (n=3). (**f,g**) FACS analysis of the CD34^{+ve} bulge stem cell compartment in K14ΔNLef1 (**f**) and K15ΔNLef1 (**g**) mice (n=3). Scale bars 50μm.

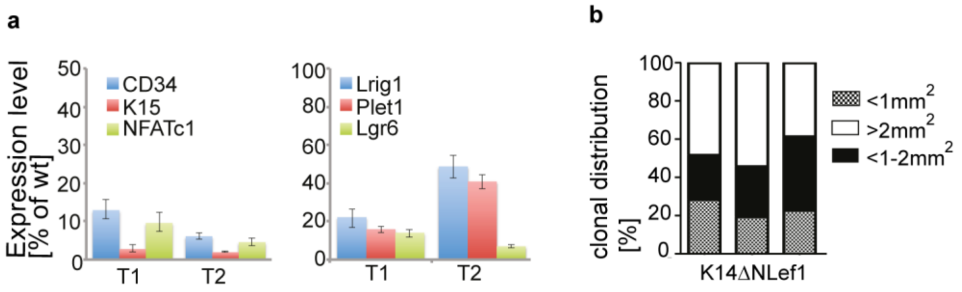

Supplementary Figure 5. Analysis of bulge stem cell marker and coloniy forming assay

(**a**) Expression of bulge stem cell marker CD34, K15 and NFATc1 (left) and JZ/I-marker Plet1, Lrig1 and Lgr6 (right) in tumours isolated from K14ΔNLef1 mice analysed by qRT-PCR. Samples were normalized to 18S and wild-type (wt) controls and SD was calculated (n=6 tumours, n=3 mice). I, isthmus; JZ, junctional zone. (**b**) Colony forming assay with primary tumour cells isolated from K14ΔNLef1 mice (n=3).

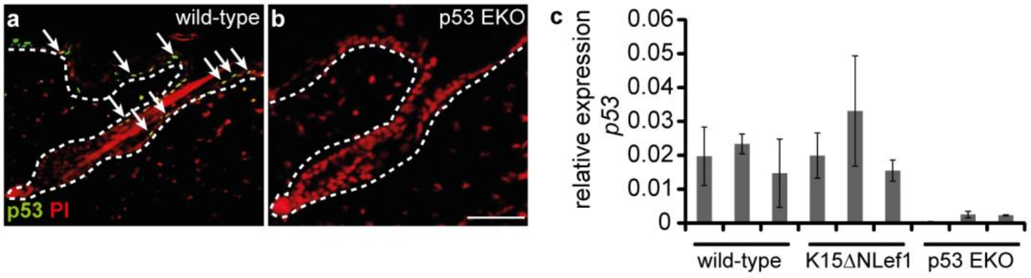

Supplementary Figure 6. Characterisation of epidermal p53 knockout mice ($p53^{EKO}$).

(a,b) Immunoflourescence staining for p53 (green, arrows) and PI (red) after UV-B irradiation in skin of wild-type (**a**) and $p53^{EKO}$ (**b**) mice (n=3). Scale bar 50µm. **(c)** qRT-PCR for p53 expression in epidermis from wild-type, K15ΔNLef1 and $p53^{EKO}$ mice. Samples were normalized to 18S and SD was calculated (n=3).

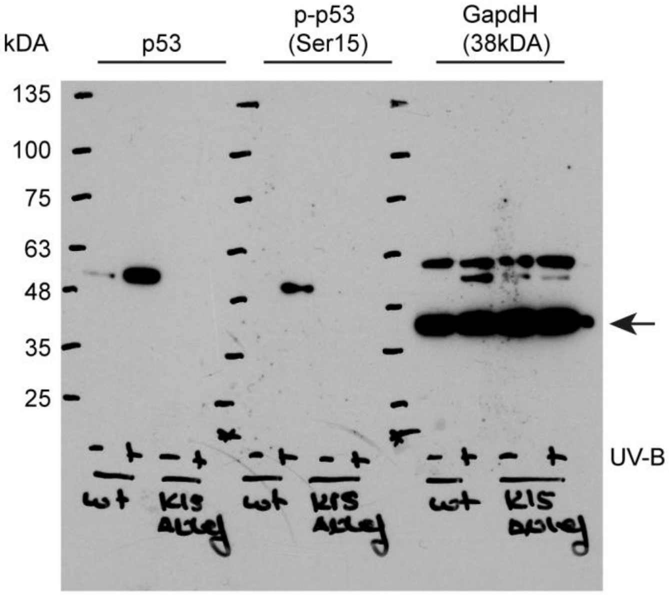

Supplementary Figure 7. p53 protein response in K15ΔNLef1 mutant mice.

Uncropped western blot for p53, p-p53 and GapDH (loading control) in epidermal lysates from wild-type and K15ΔNLef1 epidermis without (-) and 24h following UV treatment (+) (n=3).
